# Association between Veterans Aging Cohort Study (VACS) index and neurocognitive function among people living with HIV–a cross sectional study in coastal South India

**DOI:** 10.1186/s12981-021-00368-6

**Published:** 2021-08-04

**Authors:** Archana Ganapathy, Basavaprabhu Achappa, Vaman Kulkarni, Deepak Madi, Ramesh Holla, Unnikrishnan Bhaskaran, Priya Rathi, Soundarya Mahalingam, Nikhil Victor Dsouza

**Affiliations:** 1 Kasturba Medical College, Mangalore, Karnataka India; 2 Manipal Academy of Higher Education, Manipal, Karnataka India; 3 Department of Internal Medicine, Kasturba Medical College, Mangalore, Karnataka India; 4 Department of Community Medicine, Kasturba Medical College, Mangalore, Karnataka India; 5 Department of Paediatrics, Kasturba Medical College, Mangalore, Karnataka India

**Keywords:** HIV, Comorbidity, Cognition, VACS Index, CD4 cell count

## Abstract

**Background:**

HIV is an infectious disease affecting 36.7 million people worldwide. In recent times, Antiretroviral Therapy (ART) has become accessible to the majority of People Living with HIV (PLHIV) and this has transformed the course of infection to one that is chronic, characterized by fewer diseases pathognomonic of AIDS. In view of this, there is a pressing need for better markers, apart from the routine HIV indicators, to detect comorbidities such as Neurocognitive Impairment (NCI). The aim of this study was to find out the association between Veterans Aging Cohort Study (VACS) index and Neurocognitive function in HIV positive patients.

**Methods:**

In our study, we included 97 HIV positive patients and their Neurocognitive function was assessed using a combination of Montreal Cognitive Assessment and Grooved Pegboard Test, while VACS index was calculated using the most recent laboratory values. Binomial Logistics Regression analyses, adjusting for potential confounding variables, was performed to determine the association between VACS score and Neurocognitive Impairment.

**Results:**

We found that a higher VACS Index was associated with global and domain-wise Neurocognitive impairment (p < 0.01), specifically in the domains of attention (p < 0.01) and fine motor skills (p = 0.01). Our study also showed that among all the VACS components, older age (p = 0.02) and lower hemoglobin (p < 0.01) values were associated with global NCI. After plotting an ROC curve, a VACS cut-off score of 11.00 was identified as it had good sensitivity (87.0%) and specificity (71.4%) in identifying Global NCI.

**Conclusion:**

Our findings extend prior research on the use of VACS Index to predict global and domain-wise NCI in HIV-positive patients. However, further research with more comprehensive neurocognitive testing is required in our setting before VACS Index can be used as a tool to screen for neurocognitive dysfunction among PLHIV.

**Supplementary Information:**

The online version contains supplementary material available at 10.1186/s12981-021-00368-6.

## Background

HIV is an infectious disease of growing concern affecting as many as 37.6 million people worldwide with 1.5 million new infections as of end of 2020 [1]. Out of these, 27.4 million people have gained access to combination antiretroviral therapy by the end of 2020 [1, 2]. This use and accessibility of antiretroviral therapy (ART) to people living with HIV has transformed the course of infection to one that is chronic, characterized by numerous comorbid conditions, but involving fewer diseases pathognomonic of AIDS [3]. The sole use of routine HIV markers like CD4 count and viral RNA load, may not accurately reflect the impact of multisystem injury in AIDS, specifically in terms of neurocognitive disorders.

The Veterans Aging Cohort Study Index (VACS Index) is an Index which creates a score that is directly proportional to the mortality risk from all causes in HIV infected and uninfected individuals [4]. It takes into account age, the traditional HIV markers (E.g. HIV-1 RNA and CD4 count) and the general indicators of organ system injury (E.g. hemoglobin, estimated Glomerular Filtration Rate (eGFR), Fibrosis-4 (FIB-4) and viral hepatitis C infection) [4]. Recently, the use of this index was validated for estimating all-cause mortality among a large, diverse sample size with at least a year exposure to ART in North America and Europe [3].

Apart from the association of VACS Index to all-cause mortality, various studies have shown its relation to HIV related neurocognitive impairment (NCI) as well. HIV related NCI is the result of a compounding effect of HIV-1 infection and comorbidities common to HIV which cause neuropathological changes in the basal ganglia and white matter [5]. Initially in the pre-combination antiretroviral therapy (cART) era, HIV was thought to affect the subcortical areas causing striatal neurodegeneration and motor deficits. However, cortical cognitive deficits have been identified in addition to this after the introduction of cART [6]. This can be explained by poor entry of antiretrovirals into Central Nervous System (CNS) causing Cerebrospinal fluid (CSF) viral escape, permanent HIV associated neuronal damage, increased life expectancy, neurodegeneration, persistent CNS inflammation, oxidative stress and antiretroviral drug toxicity [7].

HIV Associated Neurocognitive Disorders (HAND) consists of 3 stages as per the 2007 Frascati criteria of impairment [8]. While the 3rd phase, which is the most severe form of neurocognitive impairment, has reduced following the use of ART from 7% in 1989 to 1% in 2000, the prevalence of the less severe forms have significantly increased [9]. In a south Asian study, the prevalence of HAND among people on cART was 22.7% of which 70% were Asymptomatic Neurocognitive Impairment, 23.3% were Mild Neurocognitive Disorder and 6.7% were HIV Associated Dementia (HAD) [10]. The clinical features of NCI include memory impairment, cognitive slowing, decreased fine motor functions and processing speed, all of which cause a significant drop in the capability to perform Activities of Daily Living (ADL). This in turn leads to an overall poor Quality of Life [11].

The association between a higher VACS score and HIV related NCI has been previously established in 2 cohort studies [3, 10] and a cross sectional study [12] conducted under the University of California San Diego (UCSD) HIV Neurobehavioral Research Programme where the sample size consisted predominantly of white non-Hispanic males. However, in another study performed in the rural population of sub-saharan Africa, no significant association was found between VACS Index and any stage of HIV Associated Neurocognitive Disorders as per Frascati criteria [13]. Thus, the contrasting result with respect to the significance of their association among different populations has urged us to conduct this study.

The aims of this study were to find the association between VACS index and global and domain wise NCI, following which an optimal VACS cut off score could be identified to predict NCI. The correlation of each specific cognitive domain with VACS has also been evaluated.

## Methods

### Design

This cross sectional study was carried out in a tertiary care teaching hospital affiliated to Kasturba Medical College, Mangalore, South India over a period of five months from May to September 2017.

### Setting

Since 1998, this hospital has an ART center that is dedicated to treating HIV-positive patients. It treats patients not only from its home district but also acts as a referral center for hospitals from neighboring districts and states. It provides healthcare services and drugs for treatment, along with counseling. From this center, 97 HIV-infected patients who had the necessary laboratory values required to compute the VACS Index and had valid global neurocognitive scores from the tests performed (while being free from physical or sensory problems that would interfere with neurocognitive testing) were included in the study. If participants had more than one set of data from different time periods available for VACS Index computation and Neurocognitive testing, then the most recent data was used.

The sample size was calculated based on the findings of previous studies wherein 40.6% of the HIV positive patients had impaired neurocognitive function [3]. Taking an alpha error of 5% and absolute precision to be 10% with 95% Confidence Interval, the sample size was calculated to be 97.

### Data collection and analysis

A pretested semi-structured proforma was used for the collection of sociodemographic details and past history of any comorbidity (based on clinical records) from the study participants. The comorbidities collected include hypertension, diabetes mellitus, hyperlipidemia, ischemic heart disease, cardiac failure, stroke/transient ischemic attack, thyroid disorders, Hepatitis B, and Hepatitis C. The current mood was assessed using Beck’s Depression Inventory (BDI) [3]. Participants who had a history of substance abuse, past/ present history of opportunistic neurological illness based on their clinical records or who exhibited at-least borderline clinical depression (score of ≥ 17) in the BDI were excluded from the study. Participants were considered as having ‘substance abuse disorder’ if they had a history of current or lifetime abuse of any of the following: alcohol, marijuana, opioids, methamphetamine, cocaine, sedatives or hallucinogens.

### Neurocognitive assessment

In our study, we have chosen the Montreal Cognitive Assessment and Grooved Pegboard Test to detect Neurocognitive Impairment as they are sensitive to fronto-subcortical deficits association with HIV infection [3, 14]. The reasons for using these tests have been elaborated further in the methodology section of the Supplementary data.

MOCA assesses global cognition with a high sensitivity and specificity for detecting mild cognitive impairment with a cut off score of < 26 [15]. It consists of 13 tasks measuring the following eight cognitive domains: visuospatial and executive abilities (clock-drawing, three-dimensional cube copy, alternation task adapted from the Trail Making B task and abstractions); language (naming, repetition of complex sentences and fluency); attention concentration and working memory (target detection using tapping, serial subtraction and digit forward/backward); memory (delayed recall); and orientation to time and place.

Grooved Pegboard Test is a manipulative dexterity test which was performed with both dominant and non-dominant hand in our study. A score was obtained by adding the total time (in seconds) required to fill the pegboard, the number of ‘drops’ and number of pegs placed on the board. Mean score was calculated by repeating the test two times, alternating between the dominant and non-dominant hand to take care of the practice effect, and taking an average of the two scores. The dominant hand was assumed to be the hand used by the participant to sign the consent. For the purpose of this study, we used the age-specific reference data provided with the Grooved Pegboard manual. Participants with scores of more than 1 standard deviation were classified as having impaired scores.

### VACS score computation

The VACS Index was calculated using the medical records of the patients to obtain the necessary laboratory values for age, CD4 count, Hemoglobin, FIB-4, e-GFR, and presence of Hepatitis C co-infection (all of these were routinely being tested for in the local ART center) as shown in Table 1. e-GFR was calculated using the Age and Serum Creatinine values of the patient while Aspartate transaminase (AST/SGOT), Alanine Transaminase (ALT/SGPT), platelet count and age was used for calculation of FIB-4 using the following formulation:

**Table 1:** Demographic variables, psychiatric and HIV disease characteristics of the study participants (N = 97)

Characteristics	All	Neurocognitively unimpaired N (%)	Neurocognitively impaired N (%)	*p* value
Demographics				
Age [years, M (S.D.)]^a^	43.6 (09.6)	–	–	0.04
Gender^b^				
Male [N (%)]	72 (74.2)	23 (31.9)	49 (68.1)	0.26
Female [N (%)]	25 (25.8)	05 (20.0)	20 (80.0)	
Socioeconomic status [Kuppuswamy Scale] [N (%)]^c^				
Lower	02 (02.1)	00 (00.0)	02 (100.0)	0.21
Upper lower	70 (72.2)	18 (25.7)	52 (74.3)	
Lower middle	18 (18.6)	07 (38.9)	11 (61.1)	
Upper middle	07 (07.2)	03 (42.9)	04 (57.1)	
Upper	00 (00.0)	00 (00.0)	00 (00.0)	
No. of years of education [M (S.D.)]^a^	10.9 (04.4)	–	–	0.68
Psychiatric characteristics [N (%)]				
Mood symptoms (Based on Beck’s Depression Inventory)^c^				
Normal	89 (91.8)	49 (55.1)	40 (44.9)	0.78
Mild mood disturbances	08 (08.2)	05 (62.5)	03 (37.5)	
HIV disease characteristics				
CD4 cell count [M (S.D.)]^a^	466.6 (189.2)	–	–	0.11
Nadir CD4 cell count [M (S.D.)]^a^	185 (54.1)	–	–	0.22
Diagnosed with AIDs [N (%)]^c^		–	–	
Yes	13 (13.4)	00 (00.0)	13 (100.0)	0.98
No	84 (86.6)	28 (33.3)	56 (66.7)	
Time since HIV diagnosis [M (S.D.)]^a^	08.4 (02.1)	-	-	0.67
ART status^c^				
On ART [N (%)]	95 (97.9)	27 (28.4)	68 (71.6)	0.58
Not on ART [N (%)]	02 (02.1)	01 (50.0)	01 (50.0)	
Duration of ART (years) [M (S.D.)]^a^	2.6 (1.9)	–	–	0.94
Efavirenz-based treatment regimen^b^				
N (%) On Efavirenz	49 (50.5)	14 (28.6)	35 (71.4)	0.79
N (%) Not on Efavirenz	46 (49.5)	12 (26.1)	34 (73.9)	
ART regimen [N (%)]				
TDF + 3TC + EFV	49 (50.5)	–	–	
AZT + 3TC + NVP	39 (40.2)	–	–	
AZT + 3TC + LPV/r	02 (02.1)	–	–	
AZT + 3TC + ABC	02 (02.1)	–	–	
TDF + 3TC + ABC	02 (02.1)	–	–	
TDF + 3TC + LPV/r	01 (01.0)	–	–	

Statistical tests used are as follows

*TDF* Tenofovir, *3TC* Lamivudine, *EFV* Efavirenz, *AZT* Zidovudine, *NVP* Nevirapine, *LPV/r* Lopinavir-ritonavir, *ABC* Abacavir

^a^Independent sample t test, ^b^Chi Square Test, ^c^Fischer’s exact test

FIB-4 = [Age (years) x AST level (U/L)] / [Platelet Count (109/L) x √ALT (U/L)].

### Statistical analyses

All the data from this study are presented as proportions and analyzed using SPSS v20.

Chi Square test, Fischer’s exact test and independent samples t test was performed to determine the association between demographic variables and HIV disease characteristics with Neurocognitive Impairment. To examine the relationship between VACS Index and NCI, we ran a binary logistic regression analysis between dichotomized global NCI scores and continuous VACS scores. We then ran a multivariable logistic regression analyses to adjust for demographic variables and HIV disease characteristics. The accuracy of classifying the VACS Index for NCI was determined by plotting a Receiver Operating Characteristic (ROC) curve to identify a cutoff score that would maximize sensitivity without compromising specificity.

The association between each domain of MoCA with VACS scores was found out using ordinal regression analyses. The potentially confounding demographic variables and HIV disease characteristics were then added as predictors to the model in a multivariable binary logistic regression analysis.

A chi square test was performed to explore the association between each individual component of the VACS Index with NCI. Following this, a stepwise multivariable regression using a backward selection method and *p* < 0.05 stopping rule was performed to find out which VACS Component may be most important in determining its association with Global NCI.

### Ethical approval

Ethics clearance was obtained from the institutional ethics committee, Kasturba Medical College, Mangalore.

## Results

### Baseline and HIV-disease characteristics of the study participants

Table 1 displays the baseline characteristics of the 97 participants that were included in our study. Their age ranged from 20 to 62 with a mean age of 43.62 and 74.2% of them were males. Majority of them belonged to the Upper lower Socioeconomic Status (72.2%) which was graded based on the Kuppuswamy Socioeconomic Scale [16]. The mean number of years of education among the study participants was 10.9 years. Apart from age (p = 0.04, O.R. = 1.05), none of the demographic variables were found to be significantly associated with global NCI.

While assessing the psychiatric condition by the Beck’s Depression Inventory, majority (91.8%) of the patients were normal while 8.2% had mild mood disturbances. There were no participants who had borderline clinical depression or more.

The CD4 cell count of the participants ranged from 100 to 973 and had a mean of 466.64 while the mean Nadir CD4 count was 185. Also, 13.4% of them were diagnosed with AIDS. With respect to the status of antiretroviral therapy, 97.9% of the patients were on ART, while the remaining 2.1% were newly diagnosed and had not initiated therapy yet. The mean duration for which patients were on ART was 2.6 years and majority (53.7%) were placed on Efavirenz based therapies with Tenofovir, Lamivudine and Efavirenz combination being the most popular (50.5%). None of these HIV disease characteristics were found to be significantly associated with global NCI.

Additional file 1: Table S1 shows that among personal habits, 16.1% of the participants in the study were current smokers while 31 participants were excluded from the study as a result of a history of substance abuse (alcohol, marijuana, cocaine or opioids). Also, 57.7% of the study participants were found to have comorbid conditions which include hypertension, diabetes mellitus, hyperlipidaemia, ischaemic heart disease, cardiac failure, stroke/transient ischaemic attack, thyroid disorders and Hepatitis B.

### Descriptive statistics on VACS Index and NCI

71.1% of the participants had global NCI as shown in Table 2, out of which 73.2% of them had an impaired MoCA score. 89.7% and 93.8% had impaired Grooved Pegboard Test scores performed with non-dominant and dominant hand respectively. Table 4 shows the distribution of study participants in the various domains of MoCA.

**Table 2:** Association between Global NCI and VACS Index

Variable	Frequency of impaired scores		χ 2	*p*	O.R	*C.I*
	N	%				
Global neurocognition	69	71.1	17.45	0.00	1.15	1.08–1.23
Montreal cognitive assessment	71	73.2	15.91	0.00	1.14	1.07–1.21
Grooved pegboard test non dominant	87	89.7	2.70	0.10	1.05	0.99–1.10
Grooved pegboard test dominant	91	93.8	6.95	0.01	1.35	1.08–1.69

VACS score ranged from 0 to 75 with a mean of 22.86. Table 5 shows that 83.5% of the study population were lesser than 50 years old while 42.3% had a CD4 cell count of greater than 500. 31.9% of the patients had healthy Hb levels of > 13.9 g/dL and 74.2% had good FIB-4 levels. Only 0.01% of the participants had an abnormal eGFR value of 48.2 mL/min. None of them were reported to have a Hepatitis C co-infection.

### Association between VACS Index and NCI

MoCA (χ2 = 15.915, p < 0.001, OR = 1.14, CI = 1.07–1.21) and impaired Grooved Pegboard Test score using the Dominant hand (χ2 = 6.95, p < 0.001, OR = 1.35, CI = 1.08–1.69) were found to be associated with the VACS Index.

A higher VACS score was also found to be associated with an increased likelihood of global Neurocognitive Impairment (χ2 = 17.45, p < 0.001, OR = 1.15, CI = 1.08–1.23).

Table 3 shows that after adjusting for demographic variables, the overall model was found to be statistically significant (χ2 = 38.10, df = 6, p < 0.001) and a higher VACS score continued to be significantly associated with global NCI (p < 0.001). However, gender (p = 0.63), number of years of education (p = 0.91) and socioeconomic status (p = 0.93) did not emerge as significant predictors in the model.

**Table 3:** Multivariate analysis showing association between VACS Index, demographic variables and HIV disease characteristics with Global NCI

Variable	χ 2	*p*	O.R	C.I
*VACS score and demographic variables*				
VACS Score	16.43	0.00	1.15	1.08–1.24
Gender	0.24	0.63	1.44	0.34–6.15
Socioeconomic status	0.26	0.93	0.79	0.33–1.93
Years of education	0.01	0.91	1.01	0.86–1.19
*VACS Score and HIV disease characteristics*				
VACS Score	14.08	0.00	1.11	1.07–1.23
Nadir CD4 cell count	0.01	0.97	1.00	0.99–1.01
Time since HIV diagnosis	0.90	0.35	1.10	0.91–1.33
Diagnosis of AIDS	0.86	1.00	0.94	0.92–0.98
ART Status	0.21	1.00	1.21	1.13–1.24
Duration of ART	0.42	0.52	0.86	0.54–1.36
Efavirenz-based regimen	0.55	0.45	0.61	0.15–2.57

When HIV disease characteristics were adjusted for, the overall model appeared significant (χ2 = 40.52, df = 6, p < 0.001) and a higher VACS score was significantly associated with global Neurocognitive Impairment (p < 0.001). None of the HIV disease characteristics: nadir CD4 cell count (p = 0.97), time since HIV diagnosis (p = 0.35), diagnosis of AIDS (p = 1.00), ART status (p = 1.00), duration of ART (p = 0.52) and Efavirenz-based ART regimes (p = 0.45) were found to be associated with Global Neurocognitive Impairment.

**Fig. 1:**
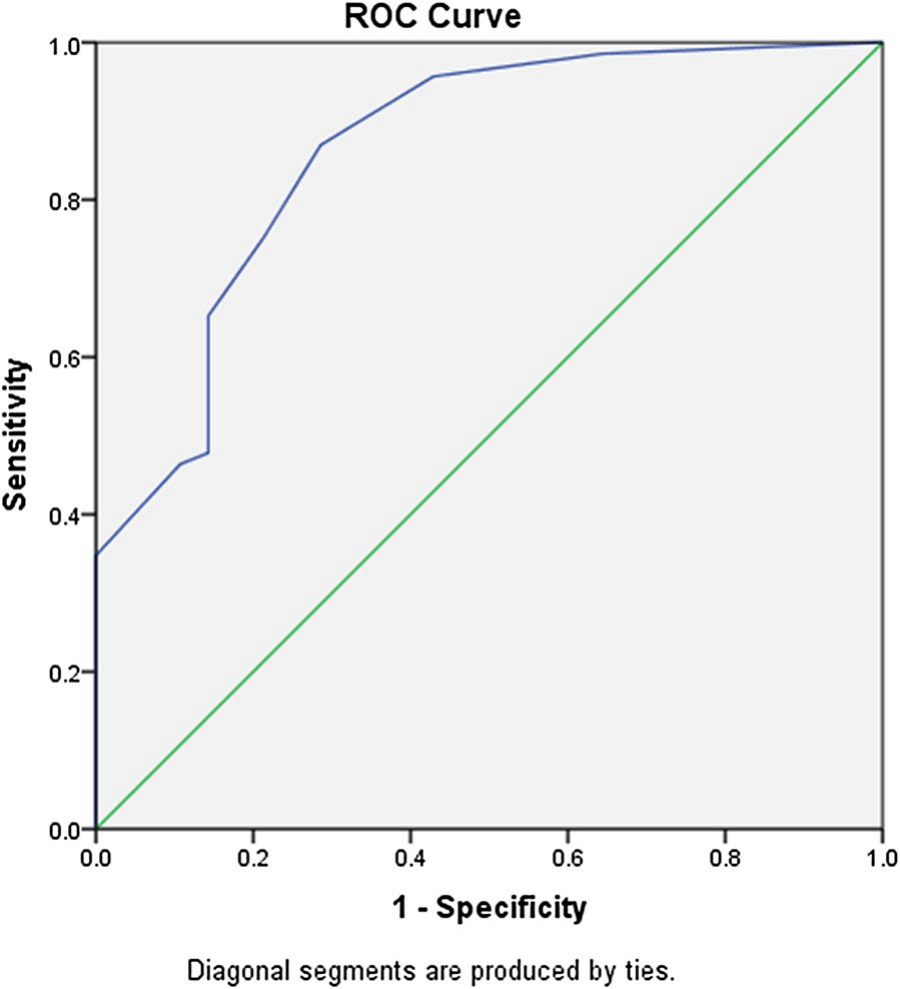
ROC Curve on association between VACS Index and Global NCI

In order to identify an optimal VACS cut off score for detecting global NCI, a Receiver Operating Curve (ROC) curve was plotted (Fig. 1). The area under the ROC curve was 0.86, 95% CI [0.78, 0.95], which is an excellent level of discrimination according to Hosmer et al. (2013). A cut-off score of 11.00 was chosen as it had a good sensitivity and specificity of 87.0% 71.4% respectively. A cut-off score of 14.00 would give a sensitivity and specificity of 75.4% and 78.6% respectively.

While performing the Montreal Cognitive Assessment, 7 domains were individually tested and the distribution of scores is presented in Table 4. The MOCA scores ranged from 11 to 28 and the mean score was 22.22 indicating that majority of the patients were neurocognitively impaired.

**Table 4:** Association between components of MoCA and VACS Index

Cognitive domain	Score	Frequencies N (%)	χ 2	*p*	O.R	C.I
Visuospatial	0	08 (08.2)	0.09	0.93	1.00	0.98–1.02
	1	04 (04.1)				
	2	06 (06.2)				
	3	14 (14.4)				
	4	22 (22.7)				
	5	43 (44.3)				
Naming	0	00 (00.0)	1.37	0.24	1.01	0.99–1.033
	1	09 (09.3)				
	2	37 (38.1)				
	3	49 (50.5)				
	4	02 (02.1)				
Attention	0	04 (04.1)	31.46	0.00	1.07	1.043–1.091
	1	05 (05.2)				
	2	10 (10.3)				
	3	14 (14.4)				
	4	21 (21.6)				
	5	24 (24.7)				
	6	19 (19.6)				
Language	0	01 (01.0)	0.18	0.67	1.00	0.98–1.02
	1	07 (07.2)				
	2	31 (32.0)				
	3	43 (44.3)				
	4	13 (13.4)				
	5	02 (02.1)				
Abstraction	0	13 (13.4)	4.38	0.04	1.02	1.00–1.05
	1	11 (11.3)				
	2	68 (70.1)				
	3	03 (03.1)				
	4	02 (02.1)				
Delayed Recall	0	40 (41.2)	0.30	0.58	1.00	0.99–1.03
	1	07 (07.2)				
	2	15 (15.5)				
	3	14 (14.4)				
	4	19 (19.6)				
	5	02 (02.1)				
Orientation	0–5	00 (00.0)	–	–	–	–
	6	97 (100.0)				

A cumulative odds ordinal logistic regression with proportional odds was run to determine the association between VACS Index and various cognitive domains assessed by MoCA. For the domain Attention, the assumption of proportional odds was met, as assessed by a full likelihood ratio test comparing the fit of the proportional odds model to a model with varying location parameters, χ^2^(5) = 1.98, *p* = 0.85. An increase in VACS score was associated with an increase in the odds of scoring 0 in the domain of Attention (χ2 = 31.46, p < 0.001, OR = 1.07, CI = 1.04–1.09).

When demographic variables (gender, years of education and socioeconomic status) were added as predictors, the overall model was significant (χ2 = 42.56, df = 6, p < 0.001) and there were proportional odds, as assessed by a full likelihood ratio test comparing the fitted model to a model with varying location parameters, χ^2^ (30) = 26.94, *p* = 0.62. There continued to be a significant effect of VACS Index (χ2 = 33.03, p < 0.001, OR = 1.08, CI = 1.05–1.10) on the domain of Attention. None of the demographic variables which were gender (p = 0.17), number of years of education (p = 0.56), or socioeconomic status categorized by the modified Kuppuswamy scale (p = 0.73) emerged as significant predictors.

HIV disease characteristics (nadir CD4 cell count, time since HIV diagnosis, ART status, duration of ART, on Efavirenz-based ART) were then added as predictors to the model and the overall model was significant (χ2 = 37.40, df = 6, p < 0.001). The assumption of proportional odds was met, as assessed by a full likelihood ratio test comparing the fit of the proportional odds model to a model with varying location parameters, χ^2^(35) = 50.60, *p* = 0.06. After adjusting for these variables, there continued to be a significant effect of VACS Index (χ2 = 28.56, p < 0.001, OR = 1.07, CI = 1.04–1.09) on the domain of Attention but there was no association between nadir CD4 cell count (p = 0.87), time since HIV diagnosis (p = 0.82), diagnosis of AIDS (p = 0.76), ART status (p = 0.20), duration of ART (p = 0.60) and Efavirenz-based ART regimes (p = 0.99) with this respective domain.

The ordinal logistics regression analyses also showed that an increase in VACS score was associated with an increase in the odds of scoring 0 in the domain of Abstraction (χ2 = 4.38, p = 0.04, OR = 1.02, CI = 1.00–1.05). The assumption of proportional odds was met for this model, as assessed by a full likelihood ratio test comparing the fit of the proportional odds model to a model with varying location parameters, χ^2^(8) = 8.62, *p* = 0.38.

After adjusting for demographic variables, (gender, years of education and socioeconomic status), the overall model was found to be significant (χ2 = 30.83, df = 6, p < 0.001) and the assumption of proportional odds was met, as assessed by a full likelihood ratio test comparing the fitted model to a model with varying location parameters, χ^2^ (18) = 1.15, *p* = 1.00. VACS Index (χ2 = 1.13, p = 0.29, OR = 0.99, CI = 1.05–1.10) and socioeconomic status (p = 0.84) were not found to be significantly associated with the domain of Abstraction but there was an association between gender (p = 0.01) and years of education (p = 0.01) with this domain. The odds of females scoring 0 in the domain of Attention was 3.76 (CI = 1.31—10.8) times that of males (χ2 = 6.100, df = 1, p = 0.01). An increase in the number of years of education was also found to be significantly associated with a decrease in the odds of scoring 0 in this domain, (χ2 = 6.72, p = 0.01, OR = 0.86, CI = 0.76–0.96).

Analyses examining the effect of HIV disease characteristics (nadir CD4 cell count, time since HIV diagnosis, ART status, duration of ART, on Efavirenz-based ART) showed that the overall model was significant (χ2 = 7.14, df = 6, p < 0.05). The assumption of proportional odds was met, as assessed by a full likelihood ratio test comparing the fit of the proportional odds model to a model with varying location parameters, χ^2^(21) = 20.18, *p* = 0.51. After adjusting for these variables, no significant association was found between the VACS score (χ2 = 3.06, p = 0.08, OR = 1.02, CI = 1.00–1.05) and the domain of Abstraction. There was no significant association between any of the HIV disease characteristics: nadir CD4 cell count (p = 0.93), time since HIV diagnosis (p = 0.09), ART status (p = 0.30), duration of ART (p = 0.21) and Efavirenz-based ART regimes (p = 0.81) with this domain either.

Table 5 depicts the components of VACS Index and the rates of global Neurocognitive Impairment in each category. The general trend shows an increase in rate of Global NCI with higher VACS scores in all categories.

**Table 5:** Univariate analysis showing association between components of VACS index and neurocognitive function

VACS component	Score	Frequency N (%)	Impaired global NCI N (%)	*p* value
Age (years)				0.03
< 50	0	81 (83.5)	54 (66.7)	
50–64	12	16 (16.5)	15 (93.8)	
> 64	27	00 (00.0)	00 (00.0)	
CD4 (cells/mm^3^)				0.08
> 499	0	41 (42.3)	26 (63.4)	
200–499	6	47 (48.4)	34 (72.3)	
100–199	10	09 (09.3)	09 (100.0)	
50–99	28	00 (00.0)	00 (00.0)	
< 50	29	00 (00.0)	00 (00.0)	
Hemoglobin (g/dL)				0.00
> 13.9	0	31 (31.9)	13 (42.0)	
12–13.9	10	27 (26.8)	20 (74.1)	
10–11.9	22	24 (25.7)	21 (87.5)	
< 10	38	15 (15.6)	15 (100.0)	
FIB-4^a^				0.06
< 1.45	0	72 (74.2)	47 (65.3)	
1.45–3.25	6	18 (18.6)	15 (83.3)	
> 3.25	25	07 (07.2)	06 (85.7)	
Egfr (mL/min)				1.00
> 59.9	0	96 (99.0)	68 (70.8)	
45–59.9	6	01 (00.0)	01 (100.0)	
30–44.9	8	00 (00.0)	00 (00.0)	
< 30	26	00 (00.0)	00 (00.0)	
Hepatitis C Co-infection	5	00 (00.0)		

*Egfr *Estimated glomerular filtration rate

^a^FIB -4: Fibrosis-4

By Univariate analysis, higher age [p = 0.034], and lower Hemoglobin [p = 0.00] were found to be significantly associated with global NCI.

Table 6 depicts the initial and final model from a multivariable stepwise regression analysis, using a backward selection method and p < 0.05 stopping rule. Both the initial (χ2 = 47.80, df = 10, p < 0.001) and final (χ2 = 40.76, df = 5, p < 0.001) models were found to be statistically significant. Age and Hemoglobin were found to be significantly associated with Global NCI in our initial model and Egfr, FIB-4 and CD4 components of the VACS score were then removed from subsequent models in that order. The final model showed that older age and lower Hemoglobin values (or higher VACS score in this category) were the strongest predictors while determining their association with global NCI.

**Table 6:** Multivariate analysis showing association between components of VACS index and Global NCI

VACS component	Initial model		Final model	
	P	OR (C.I.)	P	OR (C.I.)
Age (years)	0.04		0.02	
< 50		1.00		1.00
50–64		13.42 (1.14–158.17)		12.77 (1.38–117.92)
CD4 (cells/mm^3^)	0.90		–	
> 499		1.00		–
200–499		0.74 (0.20–2.60)		–
100–199		1.32 (1.14–2.10)		–
Hemoglobin (g/dL)	0.01		0.01	
> 13.9		1.00		1.00
12–13.9		8.94 (1.82–43.65)		5.70 (1.58–20.53)
10–11.9		21.63 (3.78–123.97)		13.24 (2.93–59.90)
< 10		24.70 (2.73–223.92)		19.21 (5.42–75.68)
FIB-4	0.15		–	
< 1.45		1.00		–
1.45–3.25		7.19 (0.98–52.65)		–
> 3.25		1.07 (0.98–1.24)		–
Egfr (mL/min)	1.00		-	
> 59.9		1.00		–
45–59.9		7.03 (1.35–36.63)		–

## Discussion

VACS Index has been proven to have a high predictive accuracy for mortality in case of HIV-infected and uninfected individuals [17] as seen in many previously conducted studies. In this particular study, we aimed to further establish an association between the VACS Index and any degree of neurocognitive impairment that maybe prevalent among People Living with HIV (PLHIV) in the Coastal South Indian population.

While analyzing the relationship between VACS Index and Neurocognitive function, it was found that higher VACS scores was significantly associated with both global and domain-wise cognitive dysfunction. The domains that were found to be most affected were attention, abstraction (assessed by MoCA) and psychomotor speed (assessed by GPT using dominant hand). The relation between Global NCI, attention and psychomotor speed with VACS Index remained significant even after adjusting for potentially confounding demographic variables and HIV disease characteristics. However, the gender and number of years of education of the participants significantly affected the association between the domain of Attention and VACS Index. It was also found that participants with higher scores in each category of the VACS Index had higher rates of Global NCI.

These results are consistent with a longitudinal cohort study conducted at the University of California [3] where VACS score was seen to cause both global and domain-wise impairment of cognitive function. Furthermore, in another study, while comparing a stably selected HIV patient population on combination ART with matched HIV uninfected controls using multivariate normative comparison to detect neurocognitive impairment, 17% of the cases were found to have impaired executive function and attention as compared to a mere 5% of the controls [18] which is in concordance with our results.

Interestingly, a cohort study conducted in the UCSD HIV Neurobehavioural Research center [19] found the difference in the specific pattern of Neurocognitive impairment in pre-Combination ART (cART) and post Combination ART era. In the pre-cART period, it was found that impairment predominated in cognitive speed, motor skills, and verbal fluency, while the cART era involved impairment of executive function (e.g. attention, planning and organizing) [20]. These results are comparable with ours where attention was found to be the significantly affected domain along with psychomotor speed in a HIV positive population where 97.9% of them were on ART.

It is important to note that there have been other studies where VACS Index has been significantly associated with impairment in all cognitive domains [3]. Here, we acknowledge the limitation of using MoCA in our study as it is currently used for level 1 diagnosis of NCI and has not been validated to assess domain-wise NCI. A study performed on 85 Parkinson’s disease patients explored the domain-specific accuracy of MoCA and found that the executive category had the highest diagnostic accuracy (84.71%) followed by attention (74.1%), language (70.6%), memory (62.4%) and visuospatial (54.12%) sections [21]. Thus, the low diagnostic accuracy and varying sensitivity/specificity of some of the sections urge the use of better tools such as complete Neuropsychological battery of tests to assess domain-wise NCI and its association with the VACS Index.

The association between older age and low Hemoglobin with NCI found in our research can be corroborated with similar findings from other studies [3, 22–24]. Age has the strongest association with NCI [3, 22] and although there is an independent additive effect of the mechanisms of HIV and aging, various longitudinal studies show significant interaction between age and HIV [25]. This association can be explained by the ubiquitous and extensive accumulation of Beta Amyloid, elevated levels of phosphorylated Tau at earlier ages in HIV positive patients, especially those on ART, and HIV induced disruption of the permeability of the Blood Brain Barrier [25]. Low Hemoglobin levels also cause higher rates of NCI as it reflects poor immunity, malnutrition of the bone marrow and decreased red cell growth factors, such as erythropoietin, which have neuroprotective effects [26].

Lower current CD4 cell counts also have been significantly associated with higher rates of neurocognitive dysfunction [6, 26–28] however, our study did not reflect this finding. This can be explained by the skewed distribution of study participants in this category. Approximately 90% of the patients had a CD4 count > 200 and none of them had a CD4 count < 100. The overall high CD4 count of the study population could be attributed to the fact that 97.9% of them were on regular ART. However, among the nine study participants who had a CD4 count of 100–199, all of them were found to have NCI. Thus, from our study findings, we cannot conclude a lack of association between lower CD4 cell counts and NCI due to skewed data distribution.

Apart from the existing components of the VACS Index, no association was found between other HIV disease characteristics such as nadir CD4 cell count, time since HIV diagnosis, AIDS defining diagnosis, ART status, duration of ART and Efavirenz based ART regimes with NCI. The consideration of non-HIV related comorbidities (e.g. cardiovascular illnesses) may improve the diagnostic utility of the VACS Index in predicting NCI. The role of additional markers to improve the predictive value of VACS Index for NCI can be further explored. For example, the addition of CD14, which is found to be significantly associated with NCI [29] and biomarkers of inflammation such as D-dimer, and Fibrin Degradation product, which increases the predictive value of VACS Index for mortality [30].

Our study has several limitations. The major weakness of this study is the use of a combination of two brief Neurocognitive tests to ascertain global Neurocognitive impairment. The rates of global NCI in other similar studies testing this association were 40% [3] and 51% [31] whereas in our study it was much higher at 71.1%. Furthermore, MoCA has not been validated for domain-wise testing of NCI, hence the associations between VACS and various cognitive domains needs to be further researched upon with comprehensive neurocognitive testing tools.

Another limitation is that HIV-1 RNA was excluded while calculating the VACS score. This is because it was not a routinely conducted test, and would be an extra financial burden for the study participants to get it tested. However, there are studies which show that even in patients not infected with HIV, who have no HIV-1 RNA (0 points) and a CD-4 count above 500 cells/mm^3^, VACS Index can reliably predict the mortality rate [32, 33]. Another study which has tried to identify the pathogenesis and prospects for treatment of HIV associated neurocognitive disorder has found that plasma HIV-1 RNA levels is not a significant risk factor for neurocognitive dysfunction, while CD4 cell count may be significant, following the introduction of Combination Antiretroviral therapy [34]. Hence, this, along with the consideration of the socioeconomic status of the population has been used as the basis for the exclusion of HIV-1 RNA in our calculation of VACS Index.

The distribution of the study participants in some categories were highly skewed. 97.9% of the participants were on ART and the other 2.1% who had not been started yet, were newly diagnosed with HIV. Although ART status did not emerge as a significant predictor while testing the association between VACS Index and NCI, this result is not reliable due to the skewed distribution of data.

There were no study participants above the age of 65 years, with CD4 count < 100 and with Hepatitis C co-infection. Furthermore, there were very few patients with evidence of liver fibrosis and only one had an egfr value < 59.9. A more even distribution of study participants in various components of the VACS score might find stronger associations between VACS Index and NCI.

Another weakness of this study is the collection of samples from a single ART Centre in Mangalore. It is not possible to project the results of this research onto the entire coastal South Indian population.

The strength of this study is that it is a novel research conducted in the South Indian population. Although there are several papers on this topic in the Western and sub-African continent, this topic has not been researched about in our study population. Through this paper, we could compare and contrast our findings with other studies and then explore the reasons behind these differences through further research. Another strength of this study is a good sample size of 97 patients which have the complete dataset required to proceed with the study.

## Conclusion

In conclusion, we have found initial evidence linking higher VACS scores to higher rates of global Neurocognitive Impairment after adjusting for demographic variables and HIV disease characteristics. A VACS cut-of score of 11.00 was found to have good sensitivity and specificity in detecting global NCI. Furthermore, the VACS components of older age and low hemoglobin were independently associated with global NCI. However, before the implementation of VACS Index as a tool to detect NCI in a clinical setting, further research is required in our study population with more comprehensive neurocognitive testing. Following this, VACS Index may function as a simple tool to help practitioners screen HIV patients who are at high risk for neurocognitive dysfunction and evaluate them further.

## Supplementary Information

**Additional file 1.** Association between Veterans Aging Cohort Study (VACS) Index and Neurocognitive Function among People Living with HIV – A Cross Sectional Study in Coastal South India.

## Data Availability

The datasets generated and/or analyzed during the current study are not publicly available due to ethical and legal reasons, but are available from the corresponding author on reasonable request.

## Abbreviations

VACS: The Veterans Aging Cohort Study Index
NCI: Neurocognitive impairment
cART: Combination antiretroviral therapy
HAND: HIV Associated Neurocognitive disorders
MoCA: Montreal Cognitive Assessment
GPT: Grooved Pegboard Test
