# Supplementary material for: Association between Veterans Aging Cohort Study (VACS) index and neurocognitive function among people living with HIV–a cross sectional study in coastal South India
Source: AIDS Res Ther. 2021 Aug 4;18:47. doi: 10.1186/s12981-021-00368-6 (PMC8336242; doi:10.1186/s12981-021-00368-6)
Supplement: Supplementary file 1 — Additional file 1. Association between Veterans Aging Cohort Study (VACS) Index and Neurocognitive Function among People Living with HIV – A Cross Sectional Study in Coastal South India. [file 12981_2021_368_MOESM1_ESM.docx]

**Supplementary Material: Association between Veterans Aging Cohort Study (VACS) Index and Neurocognitive Function among People Living with HIV – A Cross Sectional Study in Coastal South India**

**Methodology**

Montreal Cognitive Assessment and Grooved Pegboard Test have been used to detect Neurocognitive Impairment in this study. These tests have been chosen based on the results of two postgraduate theses conducted in the same setting. A study was conducted with 233 HIV participants over a 2-year period and validated the association between MoCA and Neurocognitive Impairment. MoCA yielded a high sensitivity and specificity of 78.1% and 73.9% respectively for a cut-off score of ≤ 25.5 in detecting HAND [1]. Another study used a combination of MoCA and Grooved Pegboard Test to detect NCI (measured by the Neuropsychological battery of Tests), and had a greater sensitivity and specificity of 87.5% and 76.2% respectively [2]. A MoCA cut-off score of <26 and scores of more than 1 standard deviation in GPT dominant or non-dominant were classified as having abnormal scores demonstrating performance in the well-below-average range. We have used the same criteria to determine NCI in our study.

Furthermore, the advantage of MoCA is that the test is free, available online, translated into multiple languages, has broad applicability to milder forms of HAND among the elderly in the cART era and takes a short time to perform [3]. It also allows room for correction in cases of visual impairment, physical disability or low levels of education. [4].

**Results**

**Supplementary Table 1 (S1): Baseline Characteristics of the Study Participants (N=97)**

| Variable | Descriptive Statistics |
| --- | --- |
| **DEMOGRAPHICS** |  |
| *Age (years, M [S.D.])* | 43.62 (9.564) |
| *Sex [N (%)]* |  |
| Male | 72 (74.2) |
| Female | 25 (25.8) |
| *Religion [N (%)]* |  |
| Hindu | 84 (86.6) |
| Muslim | 11 (11.3) |
| Christian | 02 (02.1) |
| *Socioeconomic Status [Modified Kuppuswamy Scale] [N (%)]* |  |
| Lower | 02 (02.1) |
| Upper Lower | 70 (72.2) |
| Lower middle | 18 (18.6) |
| Upper middle | 07 (07.1) |
| Upper | 00 (00.0) |
| *Education [Modified Kuppuswamy scale] [N (%)]* |  |
| Illiterate | 11 (11.3) |
| Primary School | 13 (13.4) |
| Middle School | 14 (14.4) |
| High School/Junior College | 34 (35.1) |
| Intermediate/Diploma | 05 (05.1) |
| Graduate | 12 (12.4) |
| Professional degree | 08 (08.3) |
| **COMORBID ILLNESSES [N (%)]** |  |
| Hypertension | 15 (15.4) |
| Diabetes Mellitus | 20 (20.6) |
| Hyperlipidaemia | 07 (07.2) |
| Ischaemic Heart Disease | 02 (02.1) |
| Cardiac Failiure | 02 (02.1) |
| Cerebrovascular Accidents | 00 (00.0) |
| Thyroid Disorders | 08 (08.2) |
| Hepatitis B | 02 (02.1) |
| Hepatitis C | 00 (00.0) |
| No comorbid illness | 41 (42.3) |

**References**

1. Srujana PSS. (2018). Human Immunodeficiency Virus Associated Neurocognitive Disorders in patients on Tenofovir+ Lamivudine+ Efavirenz (TLE) regimen [Unpublished Doctoral Dissertation]. Manipal Academy of Higher Sciences.
2. Jamshid M. (2017). Predictors of neurocognitive decline in people living with HIV on stable cART in a South Indian tertiary care center; a case control study [Unpublished Doctoral Dissertation]. Manipal Academy of Higher Sciences.
3. Valcour V, Paul R, Chiao S, Lauren A. Wendelken, and Bruce Miller. (2011). Screening for Cognitive Impairment in Human Immunodeficiency Virus. *Clin Infect Dis*, 53(8): 836–842.
4. Justice AC, Modur SP, Tate JP, Althoff KN, Jacobson LP, Gebo KA,et al. (2013). Predictive accuracy of the Veterans Aging Cohort Study index for mortality with HIV infection: a North American cross cohort analysis. *J Acquir Immune Defic Syndr*, 1;62(2):149-63
